# Imidazolines as Non-Classical Bioisosteres of *N*-Acyl Homoserine Lactones and *Quorum Sensing* Inhibitors

**DOI:** 10.3390/ijms13021284

**Published:** 2012-01-25

**Authors:** Alicia Reyes-Arellano, Alejandro Bucio-Cano, Mabel Montenegro-Sustaita, Everardo Curiel-Quesada, Héctor Salgado-Zamora

**Affiliations:** 1 Department of Organic Chemistry, National Polytechnic Institute, National School of Biological Sciences, Carpio y Plan de Ayala S/N, Colonia Santo Tomás, 11340 México, D.F., Mexico; E-Mails: abc_pp@hotmail.com (A.B.-C.); mabel_montenegro@hotmail.com (M.M.-S.); hsalgado47@hotmail.com (H.S.-Z.); 2 Department of Biochemistry, National Polytechnic Institute, National School of Biological Sciences, Carpio y Plan de Ayala S/N, Colonia Santo Tomás, 11340 México, D.F., Mexico; E-Mail: ecqmixcoacdf@gmail.com

**Keywords:** imidazoline synthesis, *quorum sensing*, *Chromobacterium violaceum*, AHL bioisosteres

## Abstract

A series of selected 2-substituted imidazolines were synthesized in moderate to excellent yields by a modification of protocols reported in the literature. They were evaluated as potential non-classical bioisosteres of AHL with the aim of counteracting bacterial pathogenicity. Imidazolines **18a**, **18e** and **18f** at various concentrations reduced the violacein production by *Chromobacterium violaceum*, suggesting an *anti-quorum* sensing profile against Gram-negative bacteria. Imidazoline **18b** did not affect the production of violacein, but had a bacteriostatic effect at 100 μM and a bactericidal effect at 1 mM. Imidazoline **18a** bearing a hexyl phenoxy moiety was the most active compound of the series, rendering a 72% inhibitory effect of *quorum sensing* at 100 μM. Imidazoline **18f** bearing a phenyl nonamide substituent presented an inhibitory effect on *quorum sensing* at a very low concentration (1 nM), with a reduction percentage of 28%. This compound showed an irregular performance, decreasing inhibition at concentrations higher than 10 μM, until reaching 100 μM, at which concentration it increased the inhibitory effect with a 49% reduction percentage. When evaluated on *Serratia marcescens*, compound **18f** inhibited the production of prodigiosin by 40% at 100 μM.

## 1. Introduction

Since the discovery of penicillin by Alexander Fleming in 1928 [1], numerous antibiotics have been introduced for the treatment of once fatal infections, many of which have spread worldwide. At first, the rapid discovery of new antibiotics led to a sufficient drug repertoire that could respond to most antibacterial requirements. However, since then the development of bacterial antibiotic resistance has become a major health issue with multifactorial causes, including the extensive and indiscriminate use of antibiotics, auto-medication, and an increased use of indwelling medical devices [2]. The latter provide a propitious environment for the growth of biofilms [2,3], which are both tenacious and highly resistant to antimicrobial treatment.

The magnitude of the problem demands new strategies. One of the options for overcoming bacterial resistance consists in interrupting a recently discovered pathway of bacterial communication [4–6], termed *quorum sensing* (QS) [7]. This communication, which regulates a variety of physiological functions, takes place through small peptides in Gram-positive bacteria, and through small molecules, such as *N*-acyl homoserine lactones (AHL, **1**) [4,5], in Gram-negative bacteria. Presumably to avoid alerting the host’s immune system to the presence of bacteria, *quorum sensing* delays virulence factor production until the cell number is high enough such that secretion of virulence factors will result in a productive infection. Therefore, the interruption of *quorum sensing* in Gram-negative bacteria in order to disable this communication system [2], through the synthesis of bioisosteres [8,9], has become a focus of research.

**Figure 1:**
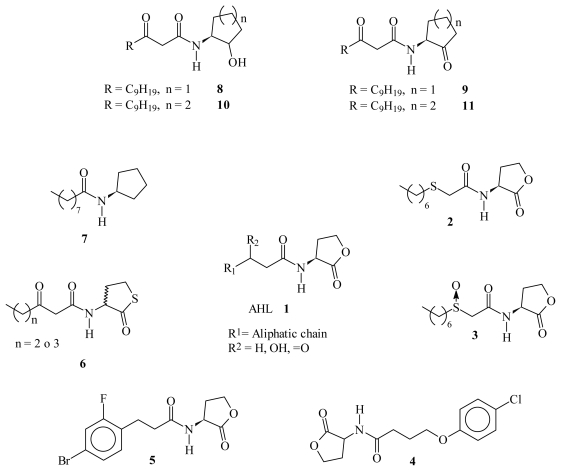
Classical bioisosteres of *N*-acyl homoserine lactones (AHL).

**Figure 2:**
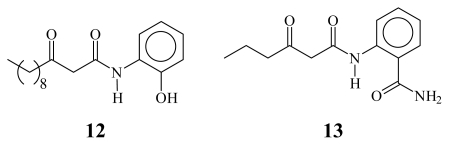
Non-classical bioisosteres of AHL.

In the search of new *quorum sensing* inhibitors against Gram-negative bacteria, AHL has been the lead compound in various studies and different strategies have been adopted. The main structural modifications on AHL to obtain antagonist and agonist bioisosteres may be summarized as follows (Figure 1): (a) Modification of the AHL aliphatic chain mediated by the introduction of an S atom (**2**) [10], SO group (**3**) [10], or a ring in the Ω carbon [11–13] (**4**, **5**); (b) Substitution of the lactone ring O atom by S [14] (**6**) or CH_2_ (**7**) [15]; and (c) Substitution of the lactone ring by another aliphatic ring (*i.e.*, cyclopentanol, cyclopentanone, cyclohexanol, cyclohexanone, *etc.*) (**8**, **9**, **10**, **11**) [16]. However, in our opinion, all of these transformations imply classical bioisosterism [8,9], and only a few examples of non-classical bioisosteres synthesis have been reported [17,18] (Figure 2).

**Figure 3:**
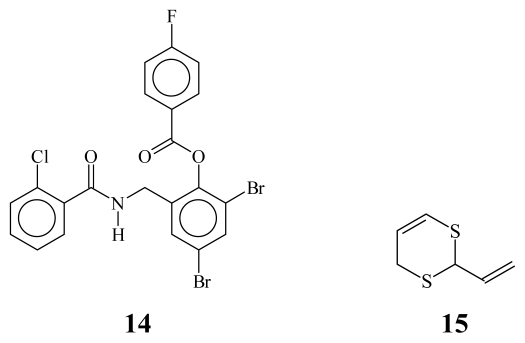
Compounds with no structural or electronic resemblance to AHLs.

Other strategies include: (a) Virtual screening of recognized drugs [19] and random screening of compounds synthesized for other purposes [20]; (b) Evaluation of plant extracts, such as the extract of *Tremella fuciformis* fruiting bodies, which showed *quorum sensing* inhibitory activity in *Chromobacterium violaceum* CV026 [21,22]. It is a fact that some positively active *antiquorum sensing* compounds do not have structural or electronic resemblance with natives AHLs [10,23], Figure 3.

The aim of the present work was to investigate a new type of non-classical bioisoster for the acyl homoserine lactone as a possible quorum sensing inhibitor. Therefore six new imidazoline derivatives were selected and evaluated as potential AHL antagonist bioisosteres [8] in the violacein production of *Chromobacterium violaceum*. Selection of *C. violaceum*, a Gram-negative bacteria, was made considering the fact that the biosynthesis of violacein, violet pigment, is triggered by *n*-hexanoyl-L-*N*-acyl homoserine lactone (C6AHL), *quorum sensing* dependent [24] and easily detectable by means of spectrophotometry.

## 2. Results and Discussion

### 2.1. Bioisosteric Design

The design of a new type of non-classical bioisosteres consisted of the substitution of the lactone ring in the acyl homoserine lactone by an imidazoline ring, this representing a bioisosteric replacement. The length of the aliphatic chain was not drastically modified, but in four of the compounds the amide functional group was replaced by an ether group. A phenyl ring was introduced to serve as a tether of the imidazoline and the aliphatic chain, with no asymmetric center. As is known, some efficient *quorum sensing* antagonists lack the asymmetric center [15]. In order to observe the electronic effects on the biological activity of *C. violaceum*, the *O*-aliphatic chain at positions *meta* and *para* on the phenyl ring was examined.

### 2.2. Chemistry

The synthesis of the imidazolines was achieved in two steps. In the first stage, the synthetic intermediates **17a**–**17d** were prepared by alkylation of 4-hydroxybenzaldehyde, and the amides **17e** and **17f** were obtained by reaction of the corresponding carboxylic acids with 4-aminobenzonitrile (Table 1). Cyclization of the required imidazolines **18a**–**18d** was achieved with ethylenediamine and iodine, while imidazolines **18e** and **18f** were synthesized with ethylenediamine and CS_2_ using MW (Table 2).

Synthetic intermediates **17a**–**17d** were prepared [25] in good yields. Amide derivative **17e** is commercially available, but both **17e** and **17f** were prepared using MW as a source of energy in an adaptation of the methodology described in the literature [26].

Imidazoline derivative **18a** has been previously prepared in 75% yield using the corresponding alkyloxybenzonitrile and inner salt *p*-CH_3_-C_6_H_4_SO_3_H_3_NCH_2_CH_2_NH_2_ [27]. Imidazoline derivative **18b** was prepared in 29% yield [28]. In contrast, these compounds and the new ones **18c**–**18d** were successfully prepared by us in quantitative yields via an adaptation of the protocol described by Fujioka [29]. Imidazoline derivatives **18e** and **18f** were synthesized in moderate yields by an adaptation of a procedure described in the literature [30]. All compounds were characterized by spectroscopic methods. The spectroscopy of known compounds in the present work was in agreement with that reported in the literature [28,31,32].

It is worth mentioning that imidazolines **18a** and **18b** were evaluated as monoxidase inhibitors with antidepressant activity, but were not highly active [28]. Imidazoline **18a** was evaluated unsuccessfully

### 2.3. Biology

#### 2.3.1. Evaluation of the Imidazoline Derivatives **18a–18f** on *Chromobacterium violaceum* wt

**Figure 4:**
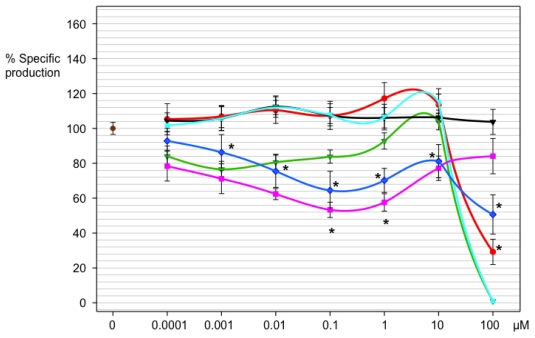
Evaluation of activity of imidazolines **18a–18f** in violacein production on *C. violaceum.* Effect of biososteres in violacein production in *C. violaceum*. Blank control (brown), **18a** (red), **18b** (green), **18c** (black), **18d** (cyan), **18e** (violet) and **18f** (blue). Asterisks indicate statistically significant activity. Data were calculated by using a two way ANOVA test with Duncan correction and are displayed as the mean ± SE (*n* = 6).

Bioisosteres of the *N*-acyl homoserine lactone, **18a–18f** were evaluated at concentrations between 100 pM and 100 μM (Figure 4). Imidazoline **18a** had an effect on the production of violacein only at a concentration of 100 μM, without affecting the growth of the bacteria. Concentrations of 25, 50, 75, 90 and 100 μM of compound **18a** were tested in order to calculate the IC50 value, which turned out to be 90.9 μM. This compound also inhibited bacteria growth at higher concentrations (data not shown). Compound **18b** did not affect the production of violacein, but it had a bacteriostatic effect at 100 μM and a bactericidal effect at a 1 mM concentration (data for the latter is not shown). The graph (Figure 4) shows an apparent inhibition between the concentrations 0.1 nM and 0.1 μM, but this effect was not statistically significant. Compounds **18c** and **18d** did not have any effect on violacein production in the time period and concentrations employed in the evaluation, except for the growth inhibition exerted by compound **18d** at a concentration of 100 μM. Bioisoster **18f** showed an inhibitory effect on violacein production starting at a concentration of 1 nM, showing an irregular performance where the highest inhibition, 49%, was observed at 100 μM (Figure 4). Compound **18e** showed a bimodal behavior, with an inhibitory effect only at 0.1 μM and 1 μM.

#### 2.3.2. Effect of Compounds **18a**–**18f** on the Viability of *Chromobacterium violaceum* Wild Type

A viable count was made of those cultures that showed inhibition of pigment production in presence of the imidazolines under study, using the concentrations at which such activity was observed. After the evaluation, it was found that the number of CFU was without change compared with the respective control group. This clearly indicated that the inhibitory effect on the production of violacein is not due to a decrease in the number of bacteria, but instead to the effects of the test compounds.

#### 2.3.3. Effects of the Imidazoline Derivatives on *Chromobacterium violaceum*

Compounds **18a**, **18e** and **18f** showed an inhibitory effect on *quorum sensing*, evidenced by the reduction in violacein production. This was confirmed by the viable count, which showed that the number of CFU was without change compared with the respective control group. Compounds **18e** and **18f** gave a linear response only at initial concentrations of 0.0001–0.1 μM. At higher concentrations, the activity was lost or reduced, suggesting that the inhibition depends on the concentration and structure of the compound. Neither **18e** nor **18f** reached a 50% inhibition under the range of concentrations tested. Although the causes of this behavior are unknown, it is possible that bioisosteres stimulate the production of the pigment at high concentrations either by inducing compensatory conformational changes in the CviR receptor, or in an indirect way, such as by stimulating the synthesis of dye precursors. A non-linear event in *QS* evaluation of certain compounds was also observed by Martinelli and collaborators [33]. They evaluated several furanones, bioisosteres of the furanones of *Delisea pulchra*, as inhibitors of the production of violacein. Martinelli concluded that the same compound may be an activator or inhibitor.

With compound **18a**, which has an ether moiety in the connector, inhibitory activity could have been the result of the imidazoline group acting as a bioisoster of the lactone ring, and the electronic conjugation of the ether with the imidazoline, conferring the latter nucleus a more basic character. Indeed, it is known that the production of violaceine is sensible to the basicity [23]. However, it is clear that basicity is not the only important feature, since imidazoline **18b**, with similar electronic properties, did not present a statistically significant *anti*-*quorum sensing* activity.

The imidazolines that have the ether group at the *meta* position (**18c** and **18d**) in the connector did not present inhibitory activity on the production of violacein, which supports the importance of the electronic conjugation. The *anti*-*quorum sensing* activity shown by the compounds **18e** and **18f** confirms that the imidazoline ring acts as a bioisoster of the lactone ring. In this case we consider that factors like the conservation of the amide group, the length of the chain, and the conjugation between the amide moiety and the imidazoline group contributed to the activity obtained. Remarkably, compound **18f** showed initial activity at the lowest concentration so far reported to inhibit *C. violaceum* QS, 1 nM.

Compound **18f** was more active than the more promising **18e**, which contains the same chain length as the acyl group in the natural AHL. Williams [34] found that as the aliphatic chain length in the majority of the AHL of Gram-negative bacteria increases, the compounds change from activators to inhibitors on the production of violacein.

The *antiquorum sensing* activity of imidazolines **18a**, **18e** and **18f** can be compared with other reports in the literature. Imidazoline **18a**, which showed the highest *antiquorum sensing* activity of all bioisosteres tested in this work (at 100 μM), rendered 71% inhibition. In contrast, 2-(4′-chlorophenoxy)-*N*-butanoyl homoserine lactone **4**, which is claimed to be the most active analog of AHL synthesized so far [11], gave a total inhibition of violacein at 10^−4^ M. The activity of compound **18a** could also be compared with some furanones, which at a concentration of 10^−4^ M showed an inhibition at around 95%, as reported by Martinelli [33].

Imidazoline **18e** showed the initial inhibition of QS at 0.1 μM (100 nM), rendering a 47% inhibition. At 1 μM this same compound yielded a 42% inhibition of the dye. By contrast, *N*-decanoyl-L-homoserine lactone (DHL) was the most active compound of a reported series of acyl homoserine lactones, showing a 51% inhibition of the dye at 100 nM [34]. Meanwhile, imidazoline **18f** showed the initial inhibition at 1 nM (the lowest concentration of this series), resulting in a 14% inhibition of QS, and at 100 μM rendered a 49% inhibition.

To summarize, imidazoline **18f** shows initial inhibitory activity of *QS* at a very low concentration (1 nM), and its highest activity (49% inhibition) at 100 μM. On the other hand, imidazoline **18a** shows the highest activity (71% inhibition) of this series at 100 μM. Thus, imidazoline **18a** and **18f** are very promising lead compounds for further research. Current structural modifications on **18a** and **18f** are under way to improve the *anti QS* activity.

#### 2.3.4. Evaluation of *N*-[4-Phenyl-(imidazo-2-yl)]-nonamide on *Serratia marcescens* ATCC 8100

**Figure 5:**
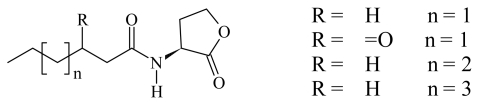
*N*-Acyl homoserine lactones utilized in *quorum sensing* by *Serratia marcescens*.

Due to the activity of compound **18f** on the production of violacein in *Chromobacterium violaceum*, this compound was evaluated on *Serratia marcescens*, a pathogenic Gram-negative bacteria found in nosocomial infections [35]. *Serratia marcescens* uses several types of signaling molecules with the *N*-acyl homoserine lactone core [36] for the synthesis of prodigiosin, a red dye that is regulated in a *QS*-dependent manner (Figure 5).

**Figure 6:**
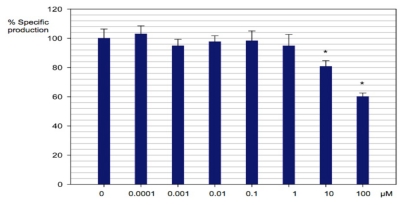
Evaluation of activity of imidazoline **18f** on *Serratia marcescens*. Data were analyzed employing the two way ANOVA test with Duncan correction, and are displayed as the mean ± SE (*n* = 6).

Compound **18f** inhibited the production of prodigiosin at practically every concentration tested (Figure 6). At 10 μM it inhibited 20% of the dye production and at 100 μM inhibited 40%. When testing other concentrations (50, 150, 200, 250 and 300 μM) of compound **18f**, the IC50 was found to be 106.5 μM. The inhibition of prodigiosin by **18f** was 15.7% at a concentration of 50 μM, 51.6% at 150 μM, 55.8% at 200 μM, 56.3% at 250 μM, and 64.8% at 300 μM. The growth of the bacteria was not affected in any case. In contrast 10 μM of nonanoyl cyclopentyl amide (**7**) rendered only 10% of prodigiosin inhibition, and 200 μM gave 85% [15].

## 3. Experimental Section

### 3.1. Experimental Chemical Section

Melting points were determined on an Electrothermal melting point apparatus and are uncorrected. Infrared spectra were recorded on a Perkin Elmer 599-B spectrophotometer. NMR spectra were recorded with a Varian Mercury 300 MHz or NMR on a Varian 500 spectrometer.

The chemical shifts (δ) are referenced to internal (CH_3_)_4_Si (δ^1^H = 0, δ^13^C = 0). The electron ionization (EI) mass spectra (70 eV) were recorded using a Hewlett Packard HP-5998A spectrometer. HRMS was determined using a JEOL-JSM-GC mateII.

*p*-Hydroxybenzaldehyde, benzonitrile and other compounds used to synthesis were purchased from Sigma-Aldrich and used without further purification except ethylenediamine, which was distillated over sodium.

#### 3.1.1. General Procedure for the Synthesis of 4-Alkyloxybenzaldehydes [25]

To a solution of 1.0 equivalent (eq) of the corresponding hydroxybenzaldehyde in acetone, K_2_CO_3_ (1.5 eq) was added and the mixture stirred for 1 h at 40 °C. Then 1.1 eq of the corresponding alkylhalide was added and the mixture was refluxed for 15 h under constant stirring. Progress of the reaction was monitored by TLC (hexane:EtOAc 8:2). Water was added to the reaction mixture and extracted with ether (3 × 20 mL). The organic extracts were combined and washed with 5% NaCl solution, dried over Na_2_SO_4_ anh. and the solvent removed under vacuum to dryness. The crude product was purified by chromatography on SiO_2_ using a gradient of a mixture hexane:EtOAc, giving the pure compounds as a colorless oil. The purified compounds were further characterized by common spectroscopic methods.

4-Hexyloxybenzaldehyde (**17a**) [31]. IR (KBr): *υ* = 2931 cm^−1^, 2858, 2734, 1695, 832; ^1^H NMR (CDCl_3_): *δ* = 9.86 (s, 1H, CHO), 7.40 (AA′BB′, 4H, Ph), 4.02 (t, 2H, OCH_2_), 1.82 (m, 2H, H-7), 1.35 (m, 6H, H-8, H-9, H-10), 0.91 (t, 3H, CH_3_); ^13^C NMR (CDCl_3_): *δ* = 190.6 (CHO), 164.1 (C-5), 131.8 (C-3), 129.5 (C-2), 114.6 (C-4), 68.2 (C-6), 31.1 (C-7), 28.9 (C-8), 25.5 (C-9), 22.4 (C-10), 13.9 (C-11).

4-Nonyloxybenzaldehyde (**17b**) [32]. IR (KBr): *υ* = 2989 cm^−1^, 2868, 2736, 1694, 830; ^1^H NMR (CDCl_3_): *δ* = 9.90 (s, 1H CHO), 7.38 (AA′BB′ 4H, Ph), 4.0 (t, 2H, H-6), 1.79 (m, 2H, H-7), 1.27 (m, 12H, H-8, H-9, H-10, H-11, H-12, H-13), 0.88 (t, 3H, CH_3_); ^13^C NMR (CDCl_3_): *δ* = 190.3 (CHO), 164.0 (C-5), 131.7 (C-3), 129.5 (C-2), 114.5 (C-4), 68.1 (C-6), 31.7 (C-7), 29.3 (C-8), 29.2 (C-9), 29.1 (C-10), 28.9 (C-11), 25.8 (C-12), 22.5 (C-13), 13.9 (C-14).

#### 3.1.2. General Procedure for the Synthesis of 3-Alkylbenzaldehydes

In a pressure tube were added 1.0 eq of 3-hydroxy benzaldehyde, 2 eq of K_2_CO_3_ and THF, and then 2.0 eq of triethylamine and 1.1 eq of alkylbromide. The reaction mixture was stirred and heated at 120 °C for 10 h (TLC SiO_2_ hexane:EtOAc 8:2). The reaction was cooled to r.t., water was added and extracted with ether 5 times. The organic phases were washed with 5% NaCl and dried over Na_2_SO_4_ anh. The solvent was removed by a rotary evaporator. The crude products were purified by chromatography on silica gel (hexane:AcOEt gradient). The pure compounds were characterized by common spectroscopic methods.

3-Hexyloxybenzaldehyde (**17c**). IR (KBr): *υ* = 2931 cm^−1^, 2858, 2725, 1698, 1263, 785; ^1^H NMR (CDCl_3_): *δ* = 9.98 (s, 1H, CHO), 7.44 (d, 2H, H-3, H-6), 7.43 (dd, 1H, H-7), 7.38 (dd, 1H, H-5), 4.0 (t, 2H, H-8), 1.80 (dt, 2H, H-9), 1.47 (m, 2H, H-10), 1.35 (m, 4H, H-11, H-12), 0.91 (t, 3H, CH_3_); ^13^C NMR (CDCl_3_): *δ* = 192.2 (CHO), 159.6 (C-4), 137.78 (C-2), 129.9 (C-6), 123.3 (C-7), 121.9 (C-5), 112.6 (C-3), 68.2 (C-8), 31.5 (C-9), 29.0 (C-10), 25.6 (C-11), 22.6 (C-12), 14.0 (C-13).

3-Nonyloxybenzaldehyde (**17d**). IR (KBr): *υ* = 3068 cm^−1^, 2925, 2854, 2724, 1701, 1262, 786; ^1^H NMR (CDCl_3_): *δ* = 9.93 (s, 1H, CHO), 7.40 (d, 2H, H-3, H-6), 7.36 (dd, 1H, H-7), 7.15 (dd, 1H, H-5), 3.97 (t, 2H, H-8), 1.79 (dt, 2H, H-9), 1.44 (m, 2H, H-10), 1.26 (m, 10H, H-11, H-12, H-13, H-14, H-15), 0.86 (t, 3H, CH_3_); ^13^C NMR (CDCl_3_): *δ* = 192.0 (CHO), 159.6 (C-4), 137.6 (C-2), 129.8 (C-6), 123.1 (C-7), 121.8 (C-5), 112.6 (C-3), 68.1 (C-8), 31.8 (C-9), 29.4 (C-10), 29.3 (C-11), 29.2 (C-12), 29.0 (C-13) 25.9 (C-14), 22.6 (C-15), 14.0 (C-16).

#### 3.1.3 General Procedure for the Synthesis of Imidazolines from Benzaldehydes

To 1 eq of alkyloxybenzaldehyde dissolved in *t*-BuOH was added 1.1 eq of ethylenediamine, at r.t. under N_2_ atmosphere and with constant stirring. The reaction mixture was held under these conditions for 1 h, then 1.25 eq of I_2_ and 3 eq of K_2_CO_3_ were added. The resulting mixture was stirred at 70 °C for 3 h. Then the mixture was filtered and the residue was poured in water and extracted with EtOAc 5 times. The organic phase was washed with a saturated solution of Na_2_SO_3_ and dried over Na_2_SO_4_ anh. The solvent was evaporated in a rotatory evaporator and the remnant residue was purified by chromatography on neutral Al_2_O_3_ (hexane:EtOAc gradient) to furnish the pure compounds.

8-Hexyloxyphenyl-2-imidazoline (**18a**). m.p. 120–121 °C (hexane:EtOAc 1:1); Lit. [27,28] 124 °C, 122–123 °C (acetone:hexane); IR (KBr): *υ* = 3084 cm^−1^, 2929, 1615, 1513, 841, 741; ^1^H NMR (DMSO-d_6_): *δ* = 7.59 (AA′BB′, 4H, Ph), 4.07 (t, 2H, H-9), 3.91 (s, 4H, H-4, H-4′), 1.73 (qi, 2H, H-10), 1.42 (m, 2H, H-11), 1.31 (m, 4H, H-12, H-13), 0.88 (t, 3H, CH_3_); ^13^C NMR (DMSO-d_6_): *δ* = 163.8 (C-2), 162.9 (C-8), 130.6 (C-6), 115.0 (C-5), 114.7 (C-7), 68.0 (C-9), 44.8 (C-4, C-4′), 30.9 (C10), 28.4 (C-11), 25.0 (C-12), 22.0 (C-13), 13.8 (C-14); MS (70 eV): m/z (%) = 246(22%) [M^+^].

8-Nonyloxyphenyl-2-imidazoline (**18b**). m.p. 107–108 °C (EtOAc:acetone 8:2), Lit. [28] 108–108.5 °C; IR (KBr): *υ* = 3217 cm^−1^, 2922, 1618, 1246, 828; ^1^H NMR (DMSO-d_6_): *δ* = 6.55 (AA′BB′, 4H, Ph), 3.22 (t, 2H, H-9), 2.78 (s, 4H, H-4, H-4′), 0.90 (qi, 2H, H-10), 0.49 (m, 10H, H-11, H-12, H-13, H-14, H-15), 0.10 (t, 3H, CH_3_); ^13^C NMR (DMSO-d_6_): *δ* = 163.1 (C-2), 160.1 (C-8), 128.5 (C-6), 122.8 (C-5), 113.8 (C-7), 67.4 (C-9), 49.4 (C-4, C-4′), 31.2 (C10), 28.9 (C-11), 28.7 (C-12), 28.6 (C-13), 28.5 (C-14), 25.4 (C-15), 22.0 (C-16), 13.9 (C-17); HRMS = C_18_H_28_ON_2_ Calcd.: 288.2202, found 288.2201.

7-Hexyloxyphenyl-2-imidazoline (**18c**). m.p. = 88–89 °C (hexane:EtOAc 6:4); IR (KBr): *υ* = 3177 cm^−1^ (NH), 2919, 2853, 1614, 1276, 851; ^1^H NMR (DMSO-d_6_, CDCl_3_): *δ* = 7.37 (m, 1H, H-10), 7.29 (m, 2H, H9, H-6), 6.98 (ddd, *J_8,9_* = 3.0 Hz, *J_8,6_* = 8 Hz, *J_8.9_* = 9.0 Hz, 1H, H-8), 3.96 (t, *J* = 6.0 Hz) 2H, H-11), 3.78 (s, 4H, H-4, H-4′), 1.76 (m, 2H, H-12), 1.43 (m, 2H, H-13), 1.33 (m, 4H, H-14,H-15) 0.89 (t, 3H, CH_3_); ^13^C NMR (DMSO-d_6_, CDCl_3_): *δ* = 164.8 (C-2), 159.2, (C-7), 131.7 (C-5), 129.4 (C-9), 118.9 (C-10), 117.8 (C-8), 112.3 (C-6), 68.1 (C-11), 31.5 (C-4), 30.9 (C-12), 29.2(C-13), 25.6(C-14), 22.6 (C-15), 13.1 (C-16); HRMS = C_15_H_22_N_2_O Calcd.: 246.1716, found: 246.1732.

7-Nonyloxyphenyl-2-imidazoline (**18d**). m.p. 82–83 °C (H:AcOEt 6:4); IR (KBr): *υ* = 3053 cm^−1^, 2926, 2854, 1615, 1265, 739; ^1^H NMR (Acetone-d_6_ CDCl_3_): *δ* = 7.35 (ddd, *J*_10,9_ = 0.5 Hz, *J*_10,6_ = 1.0 Hz, *J*_10,8_ = 2.6 Hz, 1H, H-10); 7.272 (dd, *J*_10,9_ = 0.5 Hz, *J*_8,9_ = 6.0 Hz, 1H, H-9), 7.268 (dd, *J*_10,6_ = 1.0 Hz, *J*_6,8_ = 3.4 Hz, 1H, H-6), 6.97 (ddd, *J*_8,10_ = 2.6 Hz, *J*_8,9_ = 6.0 Hz, *J*_6,8_ = 3.4 Hz, 1H, H-8), 4.01 (t, 2H, H-11), 3.66 (s, 4H, H-4, H-4′), 1.78 (m, 2H, H-12), 1.32 (m, 2H, H-13), 1.29 (m, 10H, H-14, H-15, H-16, H-17, H-18), 0.88 (t, 3H, CH_3_); ^13^C NMR (Acetone-d_6_ CDCl_3_): *δ* = 163.1 (C-2), 158.0, (C-7), 131.3 (C-5), 128.0 (C-9), 118.1 (C-10), 115.6 (C-8), 111.6 (C-6), 66.6 (C-11), 52.9 (C-4), 48.9 (C-12, C-13), 30.6 (C-14), 28.3 (C-15), 28.0 (C-16), 24.8 (C-17), 21.3 (C-18), 12.4(C-19); HRMS = C_18_H_28_N_2_O Calcd.: 288.2202, found: 288.2209

#### 3.1.4. Synthesis of *N*-4-Benzonitrile Alkylamides

Carboxylic acid (1.0 eq) was added, at 0 °C (ice bath) under N_2_ atm. with constant stirring, to 1.0 eq of DCC and 1.0 eq of 4-aminobenzonitrile dissolved in CH_2_Cl_2_. After 5 min the ice bath was withdrawn and the mixture was stirred (protected from light). The reaction was followed by TLC (silica gel and hexane:EtOAc 9:1). The reaction mixture was filtered and the solid washed with cooled CH_2_Cl_2_. The liquid phase was evaporated and the residue was purified by column chromatography over SiO_2_/K_2_CO_3_ 5% (gradient Hexane:EtOAc).

*N*-(4-Benzonitrile) hexylamide (**17e**). IR (KBr): *υ* = 3324 cm^−1^, 2220 (CN), 1681, 840; ^1^H NMR (CDCl_3_): *δ* = 9.26 (s, 1H, NH), 7.71 (AA′BB′, 4H, Ph), 2.46 (t, 2H, H-8), 1.74 (m, 4H, H-9, H-10), 1.34 (m, 2H, H-11), 0.88 (t, 3-H, H-12); ^13^C NMR (75.4 MHz, CDCl_3_): *δ* = 172.7 (C=O), 153.8 (C*ipso* NH), 151.2 (C*ipso* CN), 132.6 (C*orto* CN), 119.3 (C*orto* NH), 105.6 (CN), 37.0 (C-8), 30.9 (C-9), 24.8 (C-10), 21.9 (C-11), 13.4 (C-12); MS (70 eV): m/z(%) = 216 (<2) [M^+^], 160 (8) [CH_2_CONHC_6_H_4_CN + H^+^], 118 (100) [NHC_6_H_4_CN].

*N*-(4-Benzonitrile) nonamide (**17f**). m.p. 45–46 °C (hexane:AcOEt 8:2); IR (KBr): *υ* = 3310 cm^−1^, 3185, 2926, 2221(CN), 1651(C=O), 843; ^1^H NMR (CDCl_3_): *δ* = 9.2 (br s, 1H, NH), 7.68 (AA′BB′, 4H, Ph), 2.42 (t, 2H, CH_2_CO), 1.73 (m, 2H, H-8), 1.27 (m, 10H, H-9, H-10, H-11, H-12, H-13, H-14), 0.87 (t, 3H, CH_3_); ^13^C NMR (CDCl_3_): *δ* = 173.0 (C=O), 153.9 (C*ipso* CN), 142.2 (C*ipso* HNCO), 132.1 (C*orto* CN), 119.6 (C*orto* HNCO), 104.9 (CN), 36.7 (C-8), 32.0 (C-9), 28.4 (C-10), 28.2 (C-11), 25.8(C-12), 25.2 (C-13), 22.3 (C-14), 13.7 (C-15); MS (70 eV): m/z(%) = 258 (30) [CH_2_CONC_6_H_4_CN], 141 (24) [C_9_H_17_CO], 60 (100) [C_2_H_6_NO].

#### 3.1.5. Synthesis of *N*-[4-Phenyl-(imidazo-2-yl)]-alkylamides

*N*-(4-Benzonitrile) alkylamide (1.0 eq) in a pressure tube was added 1.0 eq of ethylenediamine and 1.0 eq of CS_2_. The mixture reaction was stirred and irradiated in a chemical MW oven at 100 Watts in a range 112–179 °C at 15 seg intervals during 5.0 min 30 seg. The reaction mixture changed from a deep yellow to pale yellow color and was purified over neutral Al_2_O_3_ with a top layer of anh. sodium sulphite (gradient of Hexane:EtOAc and finally EtOH). The solvent was evaporated and the residue was recrystallized of acetone. The pure compounds were characterized.

*N*-[4-Phenyl-(imidazo-2-yl)]-hexylamide (**18e**). IR (KBr): *υ* = 3358 cm^−1^, 2937, 2868, 1678 (C=O), 848; ^1^H NMR (CDCl_3_): *δ* = 7.20 (AA′BB′, 4H, Ph), 3.8 (s, 4H, NCH2CH2NH), 3.04 (t, 2H, H-2), 1.45 (m, 2H, H-3), 2.28 (m, 2H, H-4), 1.22 (m, 4H, H-5, H-6), 0.81 (t, 3H, H-7); ^13^C NMR (CDCl_3_): *δ* = 164.6 (N=C–NH), 154.3 (C*ipso* NHPh), 131.0 (C*orto* N=C–NH), 112.0 (C*ipso* N=C–NH), 107.5 (C*orto* NHPh), 44.3 (C-2), 31.5 (C-3), 28.8 (C-4), 26.7 (C-5), 22.5 (C-6), 14.4 (C-7); HRMS = C_15_H_21_N_3_O Calcd.: 259.1685 found: 259.1683.

*N*-[4-Phenyl-(imidazo-2-yl)]-nonamide (**18f**). m.p. 123–124 °C (acetone); IR (KBr): *υ* = 3315 cm^−1^, 2924, 1671(C=O), 846; ^1^H NMR (CDCl_3_): *δ* = 10.38 (s, 1H, CONH), 10.3 (br.s, 1H, N=C–NH), 7.83 (AA′, BB′, 4H, Ph), 3.86 (s, 4H, CH_2_CH_2_NH), 2.34 (t, *J* = 7 Hz, 2H, CH_2_CO), 1.57, (m, 2H, H-4), 1.27 (m, 10H, H-5, H-6,H-7, H-8, H-9), 0.84 (t, *J* = 7 Hz, 3H, CH_3_); ^13^C NMR (CDCl_3_): *δ* = 172.6 (C=O), 164.4 (N=C–NH), 144.4 (C*ipso* N=C–NH), 129.7 (C*orto* N=C–NH), 118.9 (C*orto* NCO), 118.5 (C*ipso* NCO), 46.0 (=NCH_2_CH_2_NH), 36.9 (C-3), 31.7 (C-4), 29.2 (C-5), 29.1 (C-6), 29.0 (C-7), 25.4 (C-8), 22.5 (C-9), 14.4 (C-10) ; HRMS = C_18_H_27_N_3_O Calcd.: 301.2154, found: 301.2153.

### 3.2. Experimental Biological Section

#### 3.2.1. Bacterial Strains

*Chromobacterium violaceum* wt. ATCC 12472 and *Serratia marcescens* ATCC 8100 were generous gifts of Dr. Graciela Castro-Escarpulli and the ENCB Medical Bacteriology Laboratory respectively.

#### 3.2.2. Evaluation of the Effect of Bioisosteres on Cell Viability

Cells from cultures exhibiting inhibition of pigment production in the presence of the imidazolines under study were washed by centrifugation and resuspended in the original volume of medium. Serial dilutions were spread onto Luria plates (*C. violaceum*) or tripticase soy agar plates (*S. marcescens*) and counted to determine the CFU per milliliter.

#### 3.2.3. Quantification of Violacein in *Chromobacterium violaceum* Wild Type

A flask containing 30 mL of autoclaved BBL thioglycolate (Becton, Dickinson, USA), supplemented with 17 mM MgCl_2_, 26 mM K_2_HPO_4_, 2.8 mM K_2_SO_4_, 3 mg/mL L-methionine and 500 ng/mL vitamin B12 (all previously sterilized by Millipore filtration) for the optimum production of violacein, was inoculated with an overnight *Chromobacterium violaceum* culture up to an optical density (OD) of 0.1 at 720 nm. Aliquots (990 μL) of culture were placed in a 2 mL tube. Then 10 μL of the compound to be tested was dissolved in sterile dimethyl sulphoxide and diluted with the culture until reaching final concentrations of 100 pM, 1 nM, 10 nM, 100 nM, 1 μM, 10 μM, 100 μM and 1 mM. DMSO alone was added to the control tubes. All tubes were sealed with Lid Bac filters and incubated at 28 °C at 900 RPM during 15 h in the Thermomixer R (Eppendorf, Germany). A 15 h period of incubation was selected as the most adequate to measure the effect of the compounds, in accordance to the results of the kinetics of violacein production.

The activity of the bioisosteres was expressed as a percentage of specific production (sp) of violacein (as shown below) in relation to the control tubes. Each experiment was performed 6 times and the results analyzed by a two way ANOVA test with Duncan correction.

$$\begin{array}{c} \text{Specific }{\text{production}}_{\text{Im}}={\text{A}}_{577}/{\text{A}}_{720}=\text{X}\\ \text{Mean sp of controls}=100\%\\ \text{Percentage of imidazoline specific production}=100-\text{X} \end{array}$$

#### 3.2.4 Quantification of Prodigiosin on *Serratia marcescens* ATCC 8100

Trypticase soy medium (5 mL) was inoculated with the *Serratia marcescens* ATCC 8100 strain. The culture was incubated at 37 °C/24 h then adjusted to a value of one of absorbance in the spectrophotometer.

Blank control and seven concentrations (100 μM, 10 μM, 1 μM, 0.1 μM, 0.01 nM, 0.001 μM, 0.0001 μM) of the compound *N*-[4-phenyl-(imidazo-2-yl)]-nonamide were placed in 8 tubes. The tripticase medium was added to the tubes and these were incubated at 22 °C during 17 h.

#### 3.2.5. Extraction and Quantification of Prodigiosin

Aliquots with 500 μL of each culture were transferred into Eppendorf tubes of 1.5 mL. After this, 500 μL of ethanol containing 4% hydrochloric acid were added. Tubes were shaken intensively with vortex and then they were centrifuged in a microcentrifuge (Hermle Z233 M-2) at 15,000 rpm for 3 min. Finally 400 μL of the red supernatant (prodigiosin) were placed in flat-bottom microplate wells and suspension was read in an ELISA reader at 540 nm. The O.D. was also determined in the ELISA reader at 600 nm, placing 400 μL of bacterial culture from each of the tubes in a microplate well.

$$\text{Specific production of prodigiosin}=({\text{A}}_{540}/{\text{A}}_{600})$$

## 4. Conclusions

Three novel non-classical bioisosteres of AHL, imidazoline derivatives **18a**, **18e** and **18f**, were found to inhibit the production of violacein, strongly suggesting an *antiquorum sensing* effect in *Chromobacterium violaceum*. The conjugation established between the heteroatom placed at the *para* position of the phenyl ring and the imidazoline moiety was a determining factor of the activity shown by the assayed compounds. The lowest concentration giving *antiquorum sensing* activity on *Chomobacterium violaceum* was 1 nM of the imidazoline conjugated with the nonanoyl group, **18f**. This compound was active at almost all tested concentrations. The highest *QS* inhibitory activity on *C. violaceum* shown by the test compounds was by imidazoline **18a** at 100 μM, without affecting the growth of the bacteria. At higher concentrations this compound did show inhibitory activity on bacterial growth. Imidazoline **18b** had no *QS* inhibitory effect on *C. violaceum*, but it had a bacteriostatic effect at 100 μM and a bactericide effect at 1 mM. Finally, imidazoline **18f**, when tested on *Serratia marcescens*, inhibited the production of prodigiosin at 10 μM and 100 μM, which strongly suggests an *antiquorum sensing* effect.

## Figures and Tables

**Table 1:** Preparation of synthetic intermediates of imidazoline derivatives.

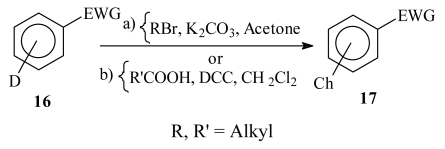					
D	EWG	Reagents and reaction conditions	Ch	Compound number	Isolated yield (%)
*p*-OH	CHO	a, reflux	*O*-nC_6_H_13_	**17a**	80
*p*-OH	CHO	a, reflux	*O*-nC_9_H_19_	**17b**	85
*m*-OH	CHO	a, reflux	*O*-nC_6_H_13_	**17c**	81
*m*-OH	CHO	a, reflux	*O*-nC_9_H_19_	**17d**	81
NH_2_	CN	b, r.t.	NHCO-nC_5_H_11_	**17e**	70
NH_2_	CN	b, r.t.	NHCO-nC_8_H_17_	**17f**	80

**Table 2:** Synthesis of the 2-arylimidazoline derivatives.

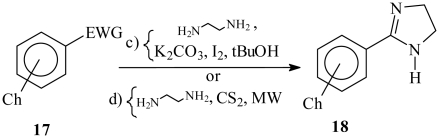				
Ch	EWG	Reagents and conditions	Compound number	Isolated yield (%)
*p*-*O*-nC_6_H_13_	CHO	c, reflux	**18a**	Quantitative
*p-O*-nC_9_H_19_	CHO	c, reflux	**18b**	Quantitative
*m*-*O*-nC_6_H_13_	CHO	c, reflux	**18c**	Quantitative
*m*-*O*-nC_9_H_19_	CHO	c, reflux	**18d**	Quantitative
NHCO-nC_5_H_11_	CN	d, MW	**18e**	50
NHCO-nC_8_H_17_	CN	d, MW	**18f**	60
